# A novel intelligent displacement prediction model of karst tunnels

**DOI:** 10.1038/s41598-022-21333-x

**Published:** 2022-10-10

**Authors:** Hai-ying Fu, Yan-yan Zhao, Hao-jiang Ding, Yun-kang Rao, Tao Yang, Ming-zhe Zhou

**Affiliations:** 1 School of Civil Engineering, Southwest Jiaotong University, Chengdu, 610031 China; 2 China Railway Eryuan Engineering Group Co. Ltd, Chengdu, 610031 China

**Keywords:** Civil engineering, Applied mathematics, Computational science

## Abstract

Karst is a common engineering environment in the process of tunnel construction, which poses a serious threat to the construction and operation, and the theory on calculating the settlement without the assumption of semi-infinite half-space is lack. Meanwhile, due to the limitation of test conditions or field measurement, the settlement of high-speed railway tunnel in Karst region is difficult to control and predict effectively. In this study, a novel intelligent displacement prediction model, following the machine learning (ML) incorporated with the finite difference method, is developed to evaluate the settlement of the tunnel floor. A back propagation neural network (BPNN) algorithm and a random forest (RF) algorithm are used herein, while the Bayesian regularization is applied to improve the BPNN and the Bayesian optimization is adopted for tuning the hyperparameters of RF. The newly proposed model is employed to predict the settlement of Changqingpo tunnel floor, located in the southeast of Yunnan Guizhou Plateau, China. Numerical simulations have been performed on the Changqingpo tunnel in terms of variety of karst size, and locations. Validations of the numerical simulations have been validated by the field data. A data set of 456 samples based on the numerical results is constructed to evaluate the accuracy of models’ predictions. The correlation coefficients of the optimum BPNN and BR model in testing set are 0.987 and 0.925, respectively, indicating that the proposed BPNN model has more great potential to predict the settlement of tunnels located in karst areas. The case study of Changqingpo tunnel in karst region has demonstrated capability of the intelligent displacement prediction model to well predict the settlement of tunnel floor in Karst region.

## Introduction

Karst mainly refers to the dissolution of soluble rocks by water and various landscape phenomena on the underground and surface^1^. Karstification often leads to rock fracture, grooves, cavities, and surface depressions that are caused by the collapse at the top of the karst cavity, which will increase the difficulty and risk of tunneling^2–7^. China is a large karst country, with a karst area of 3.44 million km^2^, accounting for about one third of the land area^8^. The karst distribution area in Yunnan Province is 110,875 km^2^, accounting for 28.14% of the total area, mainly distributed in the Northwest and Northeast^9–12^. In recent year, China has constructed a number of high-speed railway tunnels, and some of these tunnels have encountered giant karst caves^13–16^. The giant karst caves have large volume, complex geology, and high engineering risk^17–21^. Thus, the research on karst is of great significance for the scientific construction and safe operation of the tunnel.

The scale, size and location of karst have different effects on the stability of karst tunnel^12,22–24^. Except these, the stability of surrounding rock around the tunnel when the karst tunnel is located at the side, bottom and front side of the tunnel have been systematically studied, and the results indicated that the most influential position appears in the karst cave at the bottom of the tunnel^25,26^. Many studies just made a qualitative analysis like the safety thickness between tunnel floor and karst^27^, the failure mode of tunnel with different sizes and positions of karst^22–24^, but the quantitative research of maximum settlement on tunnel floor is lacking.

Forecasting the settlement can be done through either physical or data-based models^28,29^. The karst position and scale are various, making it complex to build physical models that are sufficiently representative. These shortcomings have been addressed in recent years through advances in artificial intelligence, especially machine learning algorithms, that have greatly improved modelling and forecasting of settlement^30–33^.

Recently, Machine learning (ML) techniques have recently applied in geotechnical engineering and tunnel engineering applications^34–36^. And different ML techniques have been used to solve the problem like surrounding rock deformation^37^, rock slope stability analyses^38^, ground surface settlement^39^ and other engineering problems. Arsalan Mahmoodzadeh et al.^40^ have been developed hybrid algorithms to predict Mode-I rock fracture toughness and fivefold cross-validation was applied. Besides, researchers also proposed different approaches to forecast the construction time and costs of tunneling projects, such as statistical methods^41–44^, fault tree or event tree analysis, decision trees or risk matrixes^45–47^. Arsalan Mahmoodzadeh et al.^48^ using four machine learning methods including the linear regression (LR), Gaussian process regression (GPR), support vector regression (SVR), and decision tree (DT) with the grey wolf optimization (GWO) algorithm was used to fine-tune the models' hyper-parameters to predict geology, duration, and costs of tunnels construction.

Some new back-analysis techniques have been attempted for important geotechnical problems recently. Among them, the artificial neural network (ANN), genetic algorithm (GA) and simulated annealing algorithm (SAA) are becoming popular^49,50^. In particular, the ANN including BPNN owns stronger self-adaption, self-organization, self-study and nonlinear mapping ability, which can avoid the shortcomings of traditional feedback methods and is gaining increasing popularity in recent years. The BPNN model is based on a gradient search to determine its weights, leading a tendency to fall into local minima during the learning process and significantly influenced by the initialization of the weight^51,52^. Regularization method can control the size of the network weights to effectively limit the overfitting. However, Bayes is a predictor, all predictor models have the advantage of good generalization ability. Therefore, compared with other methods, such as L_1/2_ regularization^53,54^, Tikhonov regularization^55^, Dropout regularization^56,57^ the model after Bayesian regularization has better generalization ability^58^.

The Random Forest (RF) algorithm is a new machine learning technique proposed by Breiman in 2001^59^. The RF model could effectively analyze the nonlinear, highly collinearity and mutual influence data, without assuming the mathematical form of the model in advance, which provides a new idea for predicting karst tunnel base settlement. This algorithm has been applied in biology^60,61^, soil science^62,63^, medicine^64^ et al., but the application in karst tunnel base settlement prediction is less. Machine learning algorithm with multiple hyperparameters should be optimized including RF model. At present, researchers commonly used optimization algorithm such as: genetic algorithm (GA), particle swarm optimization (PSO) algorithm and grid search (GS) algorithm. The amount of input data has a great influence on the optimization results of Genetic algorithm and swarm optimization algorithms, the grid search algorithm may not be effective^65^. Meanwhile, four kinds of common super-parameter optimization algorithm, PSO, GA, Differential evolution (DE), BO have been proposed in the process of model development for super parameter to adjust. The results show that the BO-RF model can in the shortest time and the highest accuracy forecast^65^. Therefore, the Bayesian algorithm has advantage to optimize the hyperparameters of RF that can greatly improve the efficiency of parameter regulation^66^.

According to the previous literature, a novel intelligent displacement prediction model, following the machine learning (ML) incorporated with the finite difference method, is developed to evaluate the settlement of the bottom of tunnel floor. A back propagation neural network (BPNN) algorithm and a random forest (RF) algorithm are used herein, while the Bayesian regularization is applied to improve the BPNN and the Bayesian optimization is adopted for tuning the hyperparameters of RF. The newly proposed model is employed to predict the settlement of Changqingpo tunnel floor, located in the southeast of Yunnan Guizhou Plateau, China. Numerical simulations have been performed on the Changqingpo tunnel in terms of variety of karst size, and locations. A data set based on the numerical results is constructed to evaluate the accuracy of models’ predictions. The correlation coefficients indicating that the proposed BPNN model has more great potential to predict the settlement of tunnels located in karst areas.

## The intelligent prediction model for the maximum settlement of tunnel floor

### Sources of intelligent prediction model

**Figure 1:**
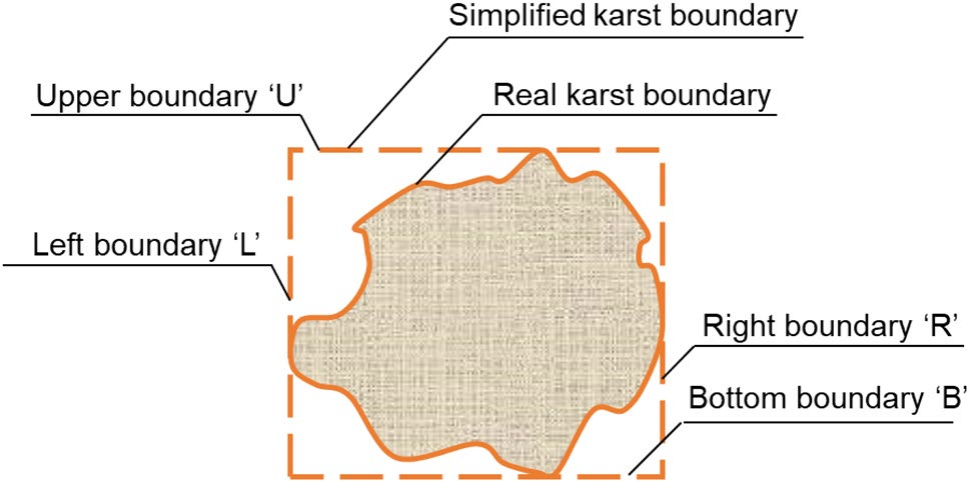
Simplified karst boundary.

The dataset being interested in is the maximum tunnel floor settlement with various karst boundary. As shown in Fig. 1, karst boundary was simplified including upper, bottom, right and left boundary, they are represented by letters U, B, R and L. The karst could at the upper, bottom, right and left of tunnel, and for each of these positions the size of karst was also considered in this paper, which could be determined by these four kinds of karst boundary. Finite difference model could provide a tunnel floor settlement with various karst boundary dataset.

**Figure 2:**
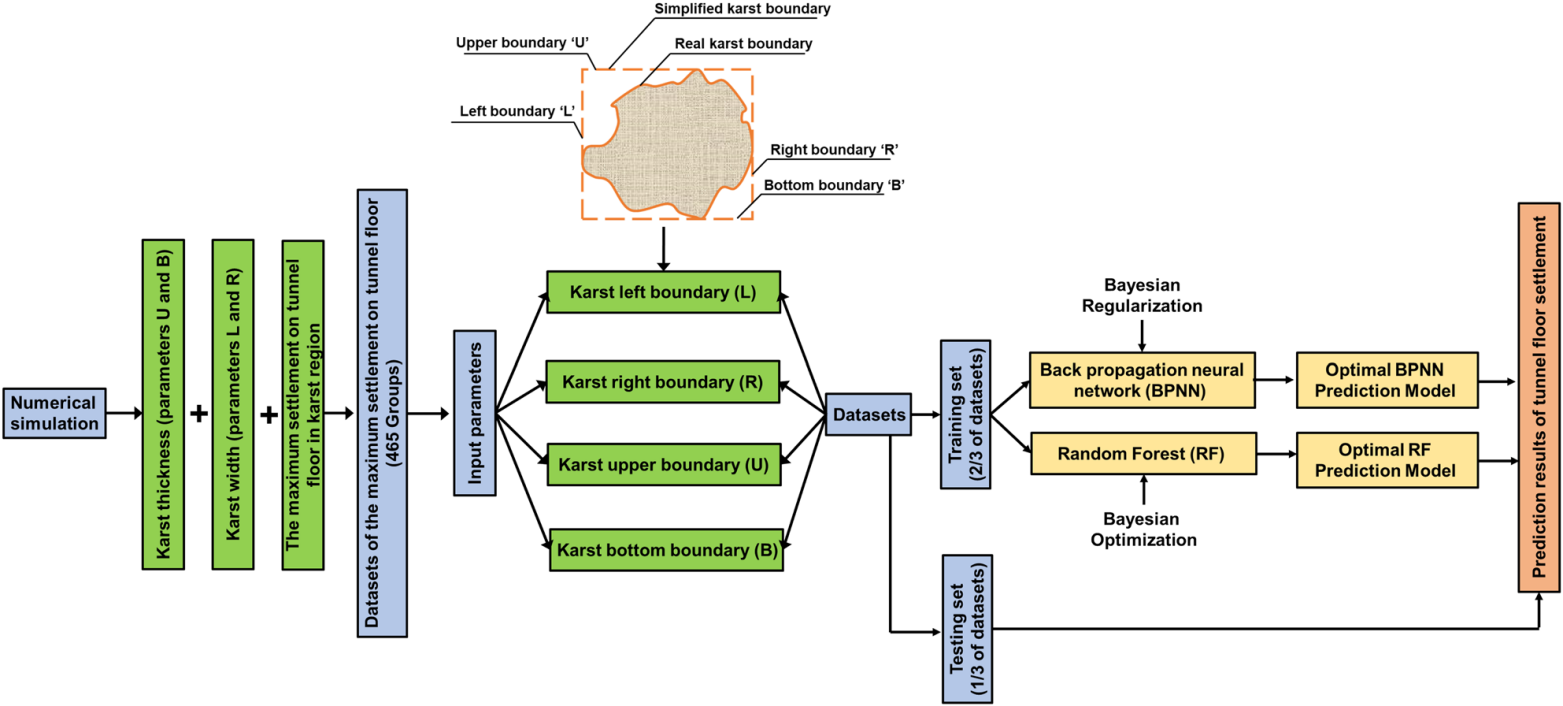
BPNN and BF model predicting process.

The predicting process on tunnel floor settlement is as follows: the dataset could be collected from the numerical simulation that analysis the influences of karst size and position on the maximum tunnel floor and corresponding karst boundary parameters to form the dataset. The karst boundary parameters are selected as the variable matrix and the tunnel floor settlement as the response matrix. Two-third the size of dataset is selected at random as the training data and the rest as the testing set. Then, Bayesian regularization was used to enhance the generalization ability of BPNN and Bayesian optimization algorithm was applied to tune RF model’s hyperparameters. Through n-time model training, a BPNN and a RF regression model are obtained, which can be used to calculate the value of the new sample in testing set. The BPNN and RF modeling process is shown in Fig. 2. And the details on the process of the optimization are presented next.

In regression analysis, mean square error (*MSE*), root mean squared error (*RMSE*), mean absolute error (*MAE*) and the correlation coefficient *r*, which are the most commonly used evaluation indicators. The *r* value is an indicator of the degree of correlation between variables. A positive *r* value means that the dependent variable increases with the independent variable, and the fitted straight line rises from left to right. The *MAE* is used to measure the average absolute error between the prediction value and the real value on the experimental data set, which cannot be used as loss functions in many cases. But *MSE* and *RMSE* could be used as a loss function, while *RMSE* is the square root of the *MSE* that has the same unit with output, which make it easy to analysis. Above all, the predictive performance of these two models could be evaluated using the root mean squared error *RSME* and the correlation coefficient *r*. And the *RSME* and *r* can be calculated by Eqs. (1) and (2):

1 $$\text{RMSE } = \sqrt{\frac{1}{{{n}}}\sum_{{{i}}= {1} }^{{n}}{\left({{y}}_{{i}}-{{x}}_{{i}}\right)}^{2}},$$

2 $$\text{r } = \frac{\sum_{{{i}}= {1} }^{{n}}\left({{y}}_{{i}}-\stackrel{\mathrm{-}}{{y}}\right)\left({{x}}_{{i}}-\stackrel{\mathrm{-}}{{x}}\right)}{\sqrt{\sum_{{{i}}= {1} }^{{n}}{\left({{y}}_{{i}}-\stackrel{\mathrm{-}}{{y}}\right)}^{2}}\sum_{{{i}}= {1} }^{{n}}{\left({{x}}_{{i}}-\stackrel{\mathrm{-}}{{x}}\right)}^{2}},$$

where *n* is the number of samples, *x*_*i*_ and *y*_*i*_ are the experimental and predicted settlement values, respectively, and $\stackrel{\mathrm{-}}{{x}}$ and $\stackrel{\mathrm{-}}{{y}}$ represent the mean values of the experimental and predicted data, respectively.

### BPNN with Bayesian regularization

**Figure 3:**
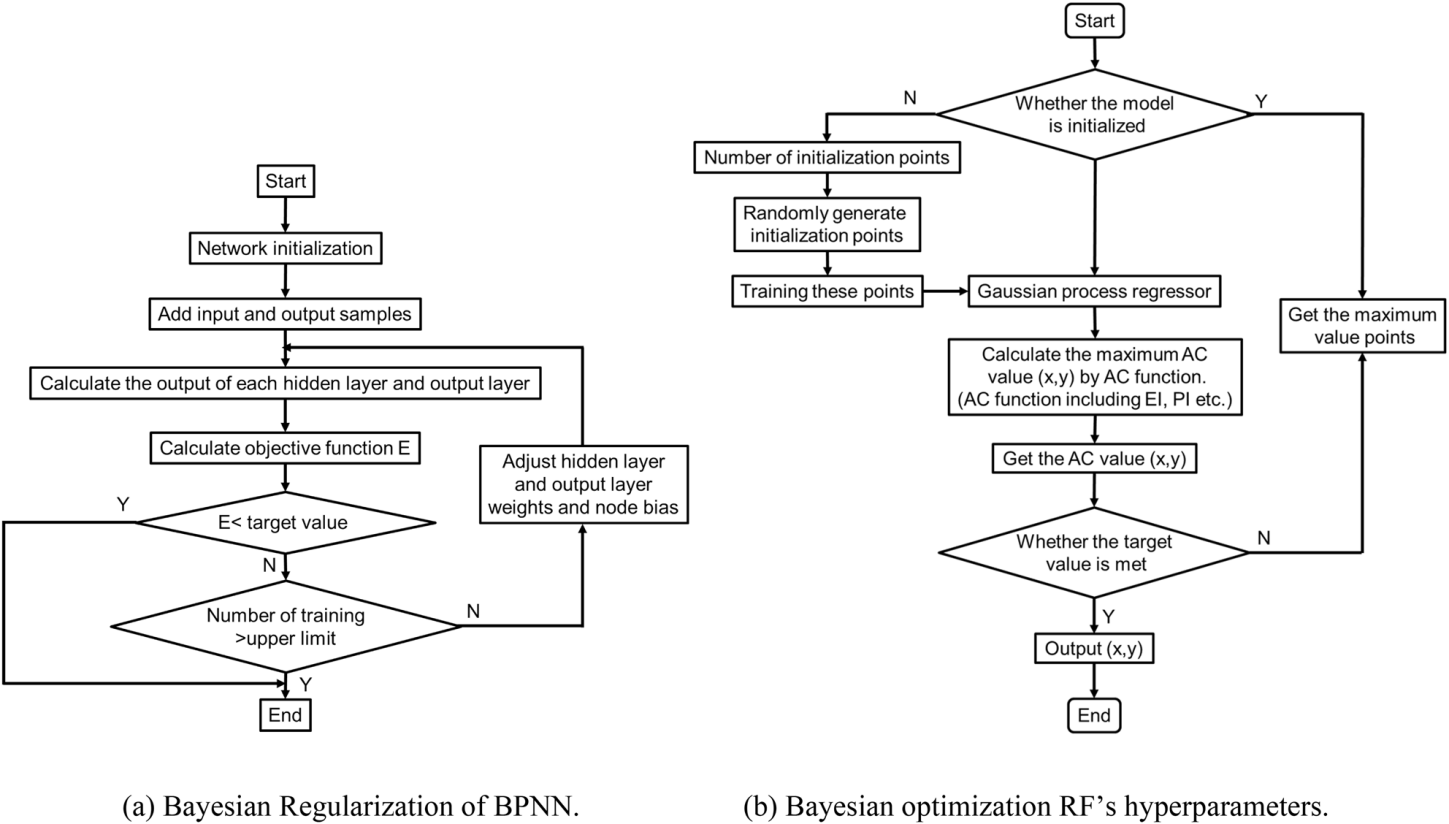
Processes of optimization.

BPNN learning algorithm was used in this study to develop a mathematical equation relating the tunnel floor settlement to karst boundary. Input parameters are the karst boundary and output the value of corresponding settlement of tunnel floor. From a trial-and-error process, ten hidden neurons and the ‘sigmoid’ transfer function were found to be the optimal architecture. The implementation process of Bayesian regularization is shown in Fig. 3a. The calculation process of tunnel floor settlement ‘s’ using BPNN is elaborated in detail as Eqs. (3) to (6):

3 $${{x}}_{{i}}{=}\frac{{2}\left({{x}}_{{actuali}}-{{x}}_{{mini}}\right)}{\left({{x}}_{{maxi}}-{{x}}_{{mini}}\right)}{-1 }\left({{i}}{=1,2,3,4}\right),$$

4 $${{A}}_{{k}}{=}{{b}}_{{hk}}{+}\sum_{{{i}}= {1} }^{4}{{{w}}}_{{hki}}{{{x}}}_{{i}} \, \left({{k}}{=1,2,3}{\ldots}{10}\right),$$

5 $${{B}}_{{k}}{=}{{w}}_{{ok}}{{{f}}}_{{sig}}\left({{A}}_{{k}}\right),$$

6 $$\text{s} = \sum_{{{i}}= {1} }^{10}{{{B}}}_{{k}}{+}{{b}}_{0}.$$

In which *x*_*i*_ is the *i*th normalized input variable. *b*_*hk*_ is the bias at neuron *k* (*k* = 1, 2, 3…14) of the hidden layer. *w*_*hki*_ is the weight connection between the neuron *k* of the hidden layer and the input variable *x*_*i*_ (*i* = 1, 2, 3 …6). *f*_*sig*_ is the sigmoid (tansig and purelin) transfer function. The ‘tansig’ was selected as the transfer function for the hidden layer to the output layer. The ‘purelin’ was selected as the transfer function for output layer to target. *w*_*ok*_ is the weight connection between the neuron *k* and the single output neuron. *b*_0_ is the bias of the output layer. *s* is the predictive result of tunnel floor settlement.

### RF with Bayesian optimization of hyperparameters

Parameter adjustment is a tedious and essential step in machine learning. Since there are many hyperparameters in every model, which together affect the accuracy of these models. Thus, manually parameter adjustment could not make the algorithm achieve the best. However, The Bayesian optimization, a mathematical method, was introduced to automatically adjust parameters in this study. The Bayesian optimization algorithm is an approximation idea that the minimum value of the objective function is found by establishing substitution function (probability model), which based on the pervious evaluation results of the objective function^67,68^. Therefore, it can be used effectively when the calculation is very complex and the number of iterations is large^69^. Compared with Grid Search^70^ and Random Search^71^, Bayesian theorem is adopted in Bayesian optimization algorithm to estimate the posterior distribution of the objective function, and then, according to the distribution, the hyperparameter combination of the next sampling was selected. The optimization is a work to find the parameters, which maximize the global result by learning the shape of the objective function. The implementation process of Bayesian optimization is shown in Fig. 3b.

As all Bayesian methodologies, it is assumed Eq. (7):

7 $${{y}}={f }{(}{{x}}{)},$$

where *f* (*x*) is a Gaussian process model that can evaluate *y*_*i*_ according to each point *x*_*i*_, which was taken at random within the variable bounds. Then repeating updating the Gaussian process model of *f* (*x*) to obtain a posterior distribution over functions *Q* (*f* |*x*_*i*_, *y*_*i*_) until finding the point *x* that maximizes the acquisition (AC) function *a*(*x*).

In terms of RF model hyperparameters, due to the algorithm generally contains one or more important hyperparameters, Bayesian optimization algorithm is adopted to automatically search the optimal solution through learning the data samples, so as to optimize the prediction effect of the model. In the RF model, the hyperparameter including: the number of decision trees (n_estimators), The minimum number of samples required to split internal nodes (min_samples_split), the number of features to consider when looking for the optimal split point (max_features)^72^.

**Figure 4:**
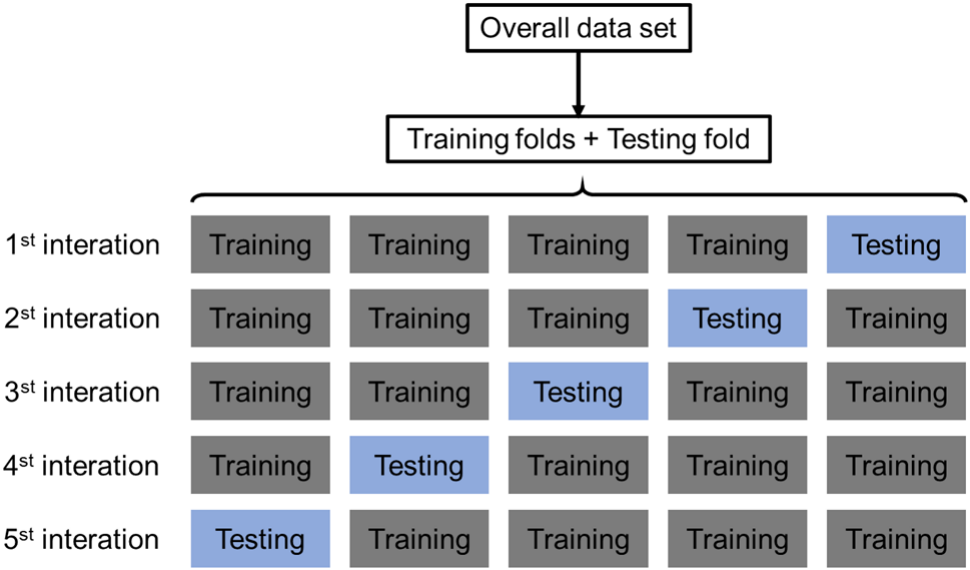
5-Fold cross-validation process.

Ideally, if the sufficient data was obtained, the best practice is to randomly divide the dataset into three parts: a training set, a validation set, and a testing set. The training set is used to fit the models; the validation set is adopted to estimate prediction error for model selection; while the testing set is utilized for assessment of the generalization error of the finalized model^73^. Since data are generally scarce, the inability to truly reflect the generalization performance of the model is common. To avoid bias in data selection, one of the most popular validation methods, such as k-fold CV^74–78^. Because k-fold cross validation can be used to evaluate the prediction performance of the model, especially the performance of the trained model on new datasets, and the over-fitting can be reduced in some degree^79^. Thus, the results of each part of the samples can be output by the K-fold cross-validation method to solve the over-fitting problem. fivefold was adopted herein (see Fig. 4).

## Changpingpo tunnel: a case study

### Engineering background

Changqingpo tunnel is in an edge slope area in the southeast of Yunnan Guizhou Plateau, connecting Puzhehei and Zhulin. The total length of the tunnel is 12.675 km. It is a double track high-speed railway tunnel. The designed clear height of the tunnel is 11.6 m and the width is 14.2 m. According to the operating speed requirements of high-speed railway trains, and the designed train operation speed is 200 km/h, and the reserved train operation speed is 250 km/h.

**Figure 5:**
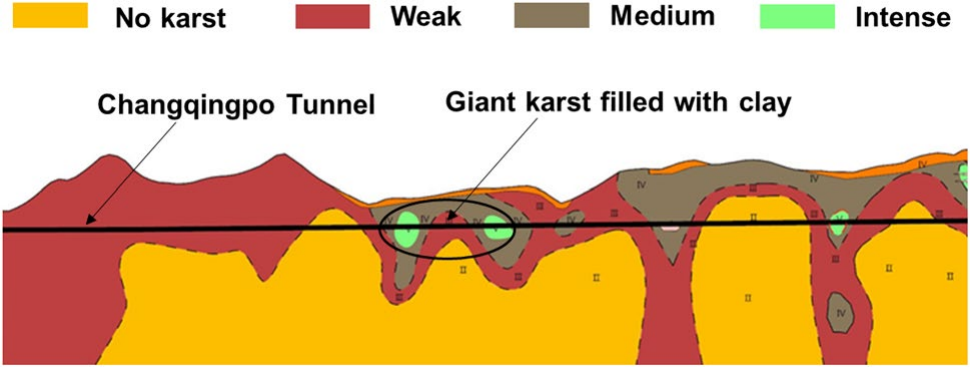
The main karst development of tunnel passing though.

There are karst fillings in the range of the lowest karst which is 43 m above the tunnel floor according to the results of drilling and exploration. According to the engineering geological exploration report of Changqingpo tunnel, the main karst development of tunnel passing though was presented in Fig. 5, blow the tunnel floor, the karst cave is filled with clay, and from the experimental and standard data, the main composition of karst fillings is clay in the consistence plastic state, which contains a small amount of breccia unevenly (about 12%) and a very small amount of gravel.

**Figure 6:**
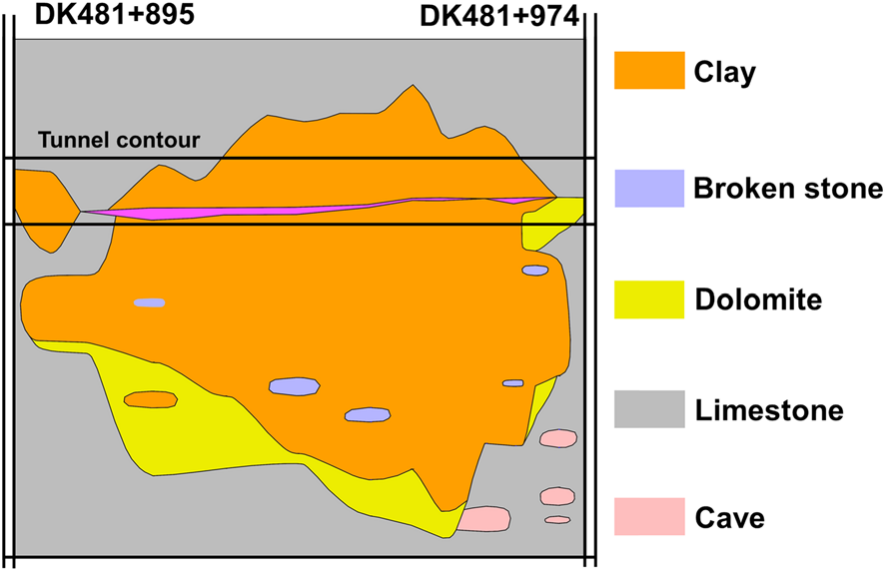
The schematic diagram of Changqingpo Tunnel.

The formation of Changqingpo Tunnel from DK483 + 978 to DK483 + 907 is mainly characterized that broken stone and clay are filled in the karst and dolomite and limestone of Upper Maping Formation (C_3_m) in Carboniferous is below the karst, and Table 1 describes the strata information. Figure 6 shows the schematic diagram of Changqingpo Tunnel of Yun-Gui Railway.

**Table 1:** The strata information of Changqingpo Tunnel from DK483 + 978 to DK483 + 907.

Strata sequence	Type	Condition	Thickness/m	Description
< 1–6 >	Filling (Q_4_^ml^)	Filling soil	0 ~ 1	Filling soil, mainly with concrete
< 2–11 >	Clay (Q_4_^ca^)	The distribution of soft and hard soil is irregular, mainly in hard plastic state, and partially in soft plastic state	0 ~ 43	The filling material of karst cave is mainly clay soil, which is gray black, yellow relatively uniform soil, and a small amount of local embedded gravel breccia
< 2–11–1 >	Broken stone	It is mainly filled with block stones	0 ~ 3	Karst cave filling material is mainly lump stone, which is gray white, and lump stone is locally distributed in clay of < 2–11 >
< 16–1 >	Limestone (C_3_m)	The joints are well developed, the dissolution erosion is serious, and the local erosion is very serious	0 ~ 16	The color of limestone is ashy, gray, which has thick layer massive structure and cryptocrystalline structure. It is the integrated contact with Weining Group below

### Model simulations

**Figure 7:**
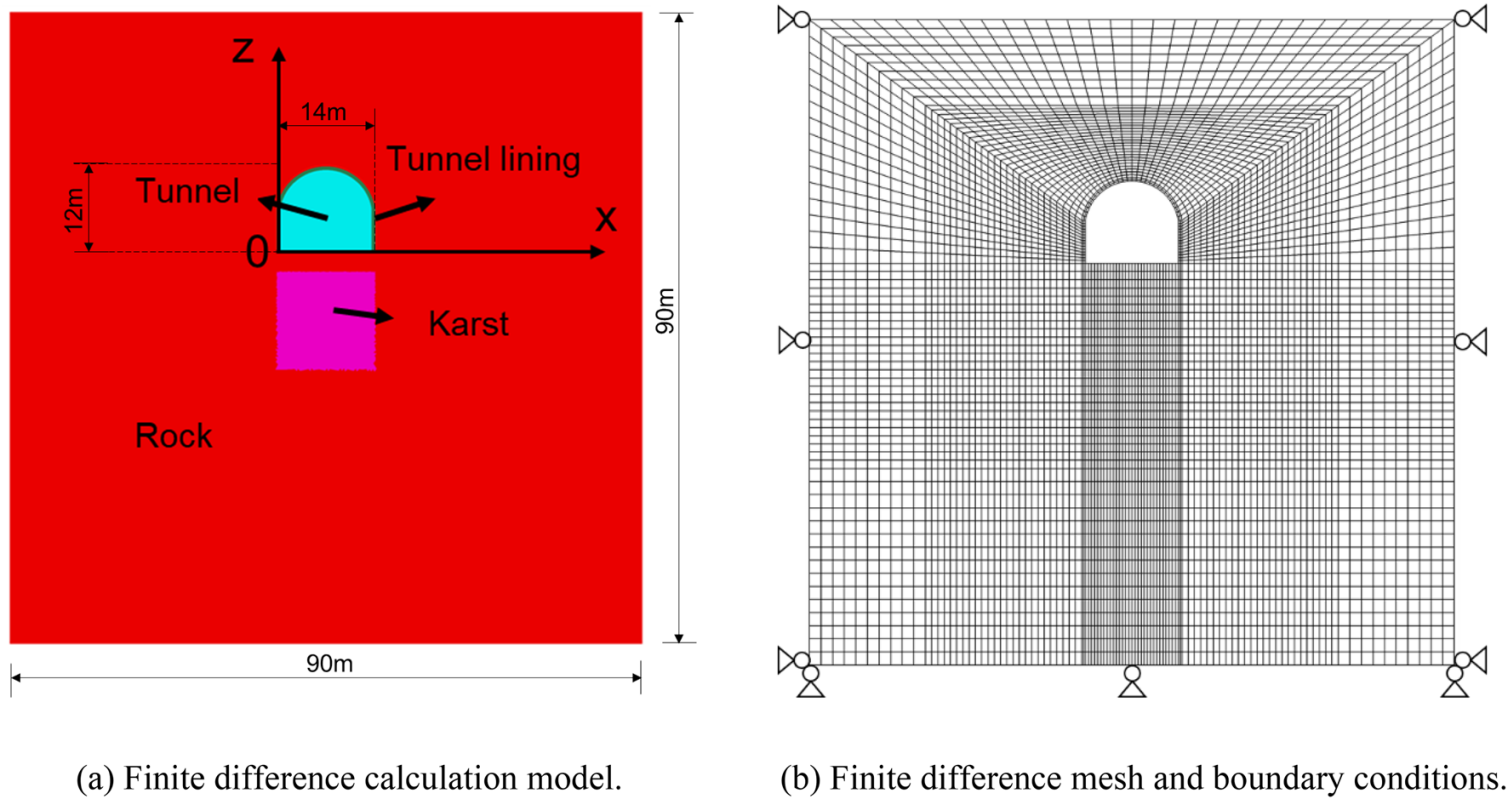
Finite difference model and mesh.

In order to eliminate the influence of boundary effect^25^, the model size is 6 times the tunnel diameter, the model dimension is 90 m × 90 m × 1 m. The tunnel adopts straight walls to connect the semicircular arch, which height is 12 m and width is 14 m, the thickness of tunneling lining is 0.5 m. Figure 7a shows the model diagram. Figure 7b illustrates the finite difference mesh for the numerical analysis, in which there are 6380 grids composed of 13,094 grid points. In the calculation model, lateral displacement boundaries were set in the X and Y direction. At the bottom of the model, the displacements in the X, Y and Z directions were restricted. The other surfaces were free.

The Mohr Coulomb constitutive model is adopted for the filling soil and rock, the tunnel lining is linear elastic constitutive model. A uniform load of 200 kN/m is applied on the tunnel floor to simulate the railway load to analyze the final settlement of the tunnel floor.

According to the report of engineering geological exploration, the karst is filling with clay, the weight, cohesion, internal friction angle, young modulus and Poisson’s ratio of the cave filling are 12.5 kN/m^3^, 23 kPa, 20°, 22.7 MPa and 0.35, respectively. The rock around the tunnel occurred mainly in dolomite and limestone deposit of Mapping Group Upper Carboniferous system, the weight, cohesion, internal friction angle, young modulus and Poisson’s ratio of the rock are 27 kN/m^3^, 1500 kPa, 45°, 7.5 GPa and 0.28, respectively. Table 2 lists the actual rock and soil mechanics parameters were selected from a field survey report.

**Table 2:** Parameter values adopted in this study.

Type	ρ (g/cm^3^)	φ (°)	c (kPa)	μ	E (MPa)	E_s_ (MPa)	K (MPa)	G (MPa)
Clay	1.35	20	23	0.35	22.7	4.54	8. 4	25.22
Rock	2.7	45	1.5 × 10^3^	0.28	7.50 × 10^3^	–	2.93 × 10^3^	5.68 × 10^3^
Tunnel lining	2.8	–	–	–	–	–	1.12 × 10^3^	3.73 × 10^3^

The variables in this numerical study can be classified into five groups by (1) effects of karst on different positions of tunnel floor; (2) location of karst; (3) karst thickness; (4) karst width; (5) quality of rock mass around the tunnel and karst.

To study the influences of karst on different positions of tunnel floor and to compare the settlement of tunnel floor with different positions, varying sites were monitoring. Altogether, 27 monitoring sites were carried out of each value of U, the details of which are given in Table 3. According to the results of effects of karst on different positions of tunnel floor, the position with the maximum settlement on tunnel floor was regarded as the monitoring site in the study of the influences of karst locations, thickness, and width. Altogether, 37 cases were carried out, the details of which are given in Tables 4, 5, and 6, respectively.

**Table 3:** Details of the effects of karst on different positions of tunnel floor.

Case	L	R	U	B	Monitoring site (x-coordinate)
1	− 38	52	2	22	0.5, 1, 1.5, 2, …, 13.5
2			3	23	0.5, 1, 1.5, 2, …, 13.5
3			4	24	0.5, 1, 1.5, 2, …, 13.5
4			5	25	0.5, 1, 1.5, 2, …, 13.5

**Table 4:** Details of the effects of karst locations on tunnel floor.

Case	Location	Distance with the tunnel/m	Thickness of karst/m	Width of karst/m
1	Up	1	14	14
2		2		
3		3		
4	Down	1	14	14
5		2		
6		3		
7	Right	1	14	14
8		2		
9		3

**Table 5:** Details of the effects of karst thickness.

Case	L	R	U	B	Thickness of karst (B–U)/m	Width of karst (R–L)/m
1	− 38	52	0	0	0	0
2			1	2, 3, 4…21	1 ~ 20	90
3			2	3, 4, 5…22	1 ~ 20	90
4			3	4, 5, 6…23	1 ~ 20	90
5			5	6, 7, 8…25	1 ~ 20	90
6			10	11, 12…30	1 ~ 20	90
7			15	16, 17…35	1 ~ 20	90
8			20	21, 22…40	1 ~ 20	90
9	0	14	3	4, 5…43	1 ~ 40	14
10			5	6, 7…45	1 ~ 40	14
11			10	11, 12…50	1 ~ 40	14
12			15	16, 17…55	1 ~ 40	14

**Table 6:** Details of the effects of karst width.

Case	L	R	U	B	Thickness of karst (B–U)/m	Width of karst (R–L)/m
1	− 5	− 4, − 3…52	1	5	4	1 ~ 57
2	− 2	− 1, 0, 1…52				
3	0	1, 2, 3…52				
4	2	3, 4, 5…52				
5	3	4, 5, 6…52				
6	5	6, 7, 8…52				
7	6	7, 8, 9…52				
8	7	8, 9…52				
9	8	9, 10…52				
10	9	10, 11…52				
11	10	11, 12…52				
12	12	13, 14…52				

Besides these, the Rock mass quality Q of karst region was also considered. Additionally, it should be noted that the Q value cannot be directly used in the numerical calculation. The rock mass mechanical parameters are determined from the Q value by mean of the empirical relationship listed in Table 7. The values of Q were selected and its corresponding mechanical parameters to be used in the numerical analysis. Altogether, 10 cases were carried out, the details of which are given in Table 8.

**Table 7:** Empirical equations relating Q with rock mass properties.

Parameters	Relationship	References
Q	Q	
RMR	RMR = 7lnQ + 36	Tugrul (1998)^80^
Cohesion (kPa)	c = 5 × (RMR − 1)	Bieniawski (1989)^59,81^
Internal friction angle φ (°)	φ = 0.5RMR + 4.5	Bieniawski (1989)^59,81^
Young’s modulus E (GPa)	E = 2 × RMR − 100 (RMR > 50)	Bieniawski (1978)^82^, Serafim and Pereira (1983)^83^
	E = 10^(RMR − 10)/40^ (RMR ≤ 50)	Bieniawski (1978)^82^, Serafim and Pereira (1983)^83^

**Table 8:** Mechanical parameters of rock mass with different Q values.

Case	Q value	Young’s modulus E (GPa)	Cohesion (kPa)	Internal friction angle φ (°)	Poisson ratio μ
1	0.1	1.77	94.41	14.44	0.40
2	0.5	3.38	150.74	20.07	0.38
3	1	4.47	175.00	22.50	0.37
4	2	5.91	199.26	24.93	0.36
5	5	8.54	231.33	28.13	0.35
6	10	11.30	255.59	30.56	0.33
7	20	14.94	279.85	32.99	0.25
8	40	19.75	304.11	35.41	0.24
9	60	29.32	318.30	36.83	0.22
10	80	33.35	328.37	37.84	0.20

### Parameter analysis

#### Validation of finite difference method

**Figure 8:**
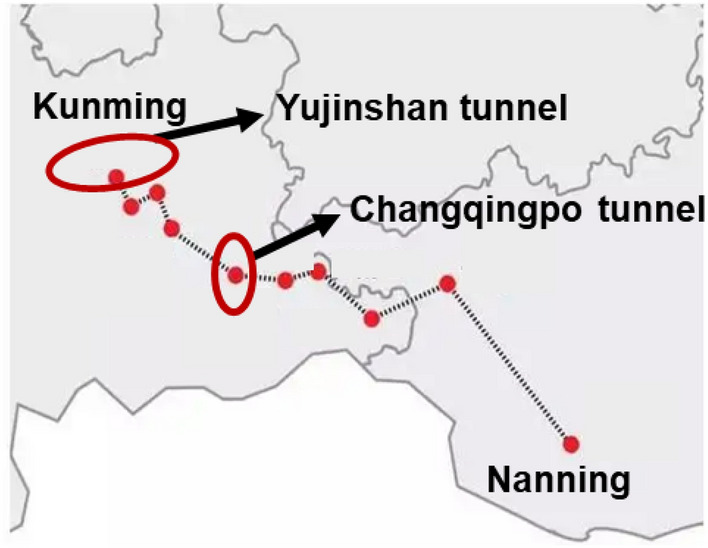
The relative location of Changqingpo tunnel and Yujinshan tunnel.

**Figure 9:**
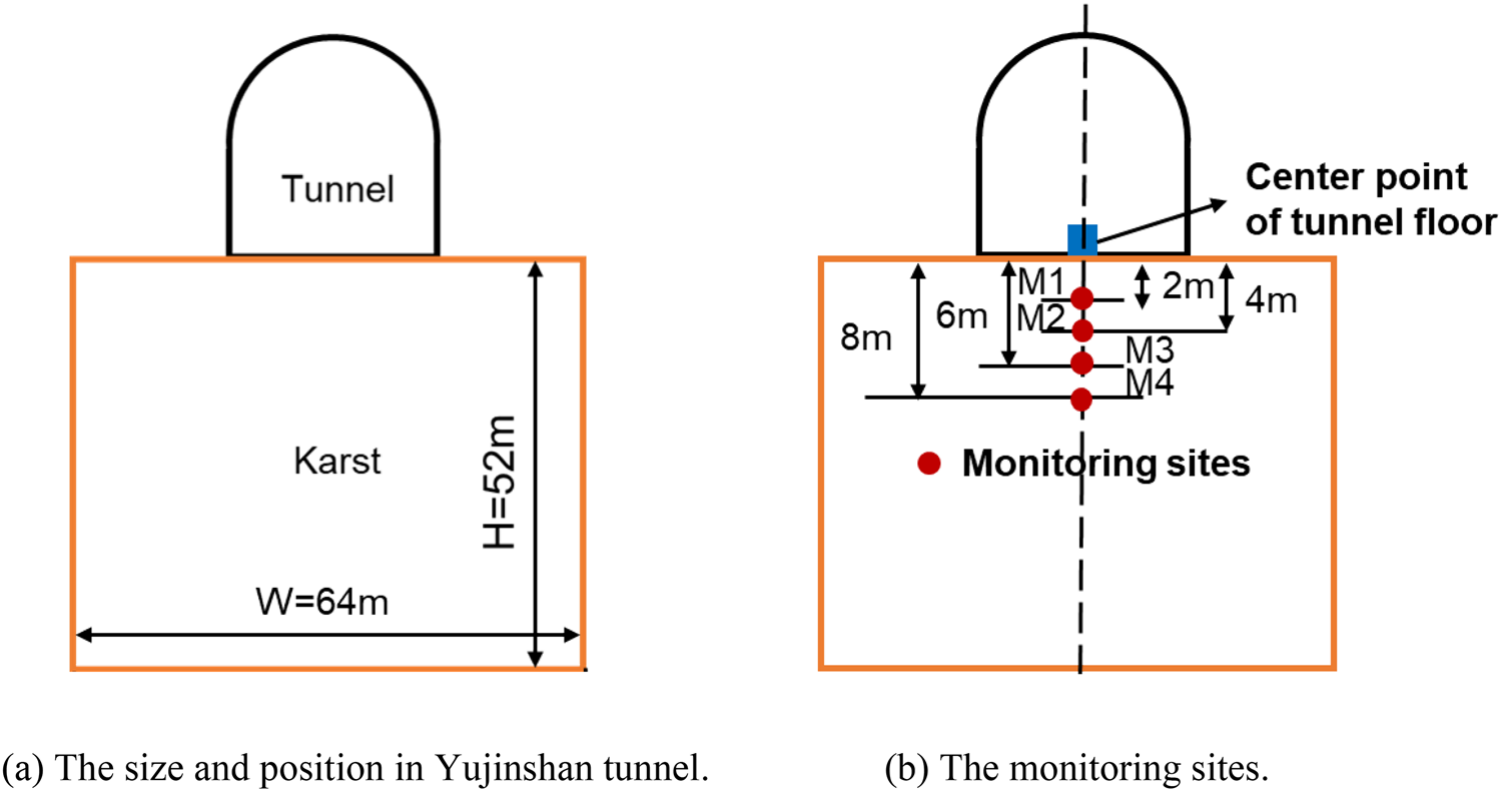
The numerical model and monitoring sites.

The validation of finite difference method was demonstrated through modeling the near engineering of Yujinshan tunnel^84^, which is in the northeast of Yunnan Province and has the same size of tunnel. The relative location of Changqingpo tunnel and Yujinshan tunnel is shown in Fig. 8. The material models and size of tunnel are presented is same as that given in Table 2. To achieve a calculate of tunnel floor settlement, a load due to machine drilling is 200 kPa was applied to the tunnel floor and the value of karst upper boundary is zero, also the thickness and the width of karst is 52 m and 64 m respectively as presented in Fig. 9a. The monitoring sites are shown in Fig. 9b.

**Figure 10:**
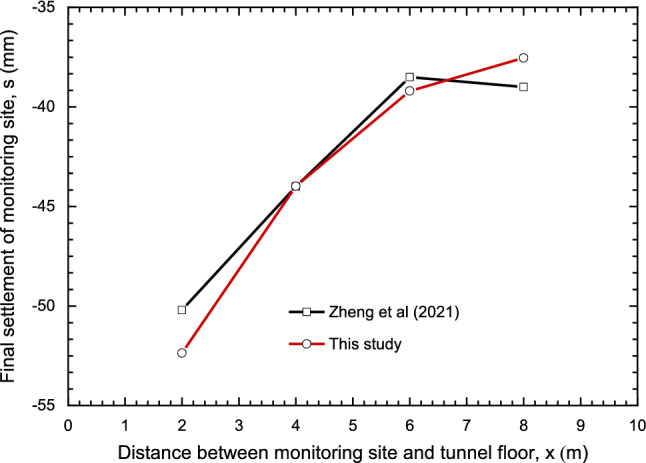
The numerical model and monitoring results.

The result was compared with the monitoring data as shown in Fig. 10. Good agreement was obtained as presented in Fig. 10. Note that due to the influence of the grouting hole at the bottom of the tunnel and the rolling back and forth of construction vehicles, the final settlement decreased with the increase of depth in both two curves, and the maximum settlement of these two curves was 50.2 mm and 52.36 mm respectively on the M1, while the minimum settlement of these two curves was 39 mm and 37.54 mm respectively on the M4. Beside these, the value of settlement in Zheng et al. is smaller than that in finite difference method, and on the contrary, the settlement in Zheng et al. is larger. Note that the relative error is in a range of 0.045–4.3%, so the resulting value of settlement produced by the finite difference method were acceptable.

#### Effects of karst on different positions of tunnel floor

**Figure 11:**
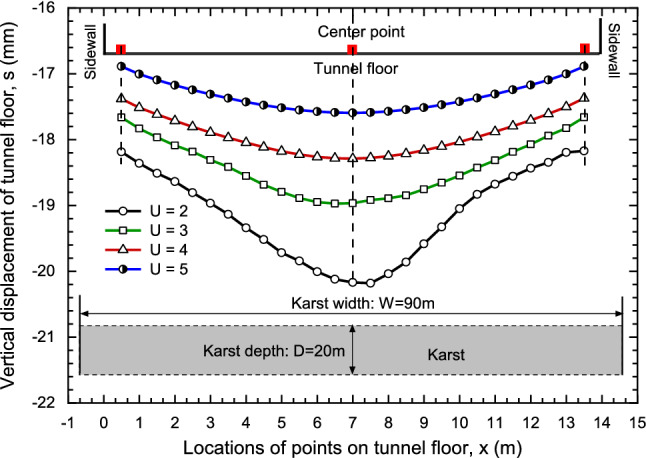
Tunnel floor settlement at different locations.

The karst located on the bottom of tunnel was firstly considered. For the giant karst cave, in order to study the effect of karst depth, the karst width was 90 m, which made left and right boundaries of karst coincided with model boundaries, considering the difference of karst thickness and upper boundary of karst, analyzing the influence of karst and the distance between tunnel floor and karst cave on the settlement of tunnel floor. The calculation results are shown in Fig. 11.

The settlement with different locations on tunnel floor was studied with varieties values of U. Considering that the karst thickness remains unchanged at 20 m and value of U is in the range of [2, 5], the settlement at different positions of tunnel floor was calculated. As shown in Fig. 11, the curve is in "V" shape, and the settlement increases from the corner of the tunnel to the middle. The settlement at the center is the largest, while at the corners are smaller than their neighbors. With the increasing of U, all curves tend to be gentle, and the settlement at the center of tunnel floor decreases gradually, which means the effect of karst gradually weakens.

#### Effects of karst position

**Figure 12:**
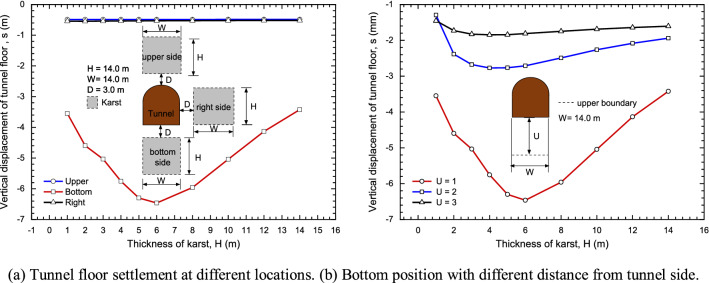
The influences of karst positions.

In order to study influences of karst’s positions, three locations including upper, bottom and right of the tunnel were calculated respectively. The karst size remains 14 m × 14 m, and the karst center coincides with the tunnel centerline. Meanwhile, the influence of settlement at tunnel floor center with varieties distance between the karst boundary and the tunnel was researched, including U = 1, 2 and 3 respectively. As shown in Fig. 12.

From the Fig. 12a, When the karst is located at the bottom of tunnel floor, the influence on the settlement is obvious, while the karst is located on right and top of the tunnel, the effect is weak^25,26^. At the same time, when the karst thickness remains unchanged, the settlement decreases gradually with the distance of karst and tunnel. If the karst is located below the tunnel floor, the curve presents a "V" shape as shown in Fig. 12b. With the increase of the karst thickness, the settlement first increases until thickness is 6 m and then decreases. With the growing of U, the settlement decreases gradually, and the curve tends to be flat.

#### Effects of karst thickness

**Figure 13:**
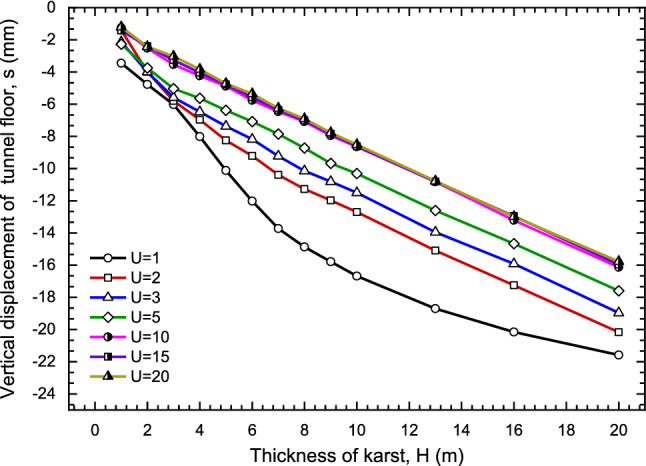
The effect of giant karst depth on tunnel floor settlement.

The settlement of the center of tunnel floor was calculated including U = 2, 3, 5, 10, 15 and 20 while the karst thickness is a value in the range of (0, 20] and the width of karst is 90 m. It can be seen from Fig. 13 that when the width of karst is huge, the settlement of tunnel floor increasing with the growing of karst thickness. Considering the influence of U is in the range of [− 5, − 1], the curve presents a concave shape. With the increase of U, the curve tends to be a straight line. When U = 15 and U = 20, curves are basically coinciding, and the influence of U on the tunnel floor settlement could be ignored. In other words, if the karst thickness is unchanged, the effect of U on the tunnel floor settlement cannot be considered while U ≥ 15, which U is two times larger than excavating radius^85^.

**Figure 14:**
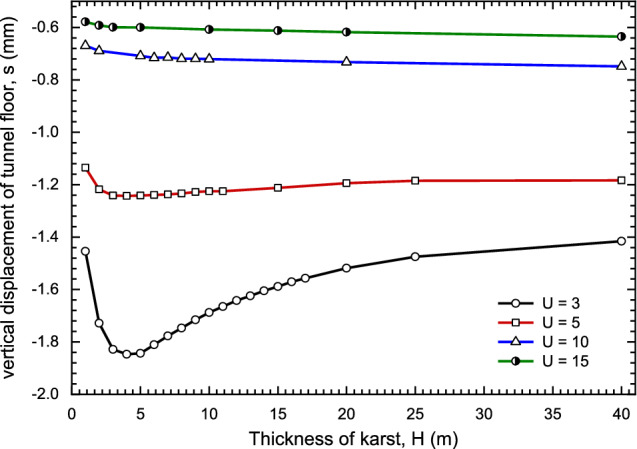
The effect of karst thickness and upper boundary.

**Figure 15:**
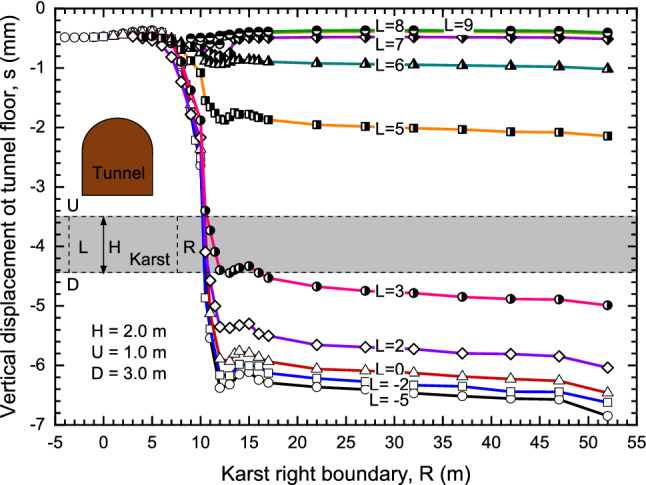
The effect of karst width (L and R) on tunnel floor settlement.

Then, for the usual size of karst (karst width is 14 m) as shown in Fig. 14, the influence of karst thickness become slight if karst thickness is larger 5 m. The impacts of U and B on the settlement were also analyzed. Under this condition, left boundary (L = 0) and right boundary (R = 13) are fixed, the relationship between karst thickness and settlement with different U is described in Fig. 15. With the decreasing of U, the settlement decreases gradually, and the settlement increases first and then decreases with the growth of thickness. Especially U = 10 and 15, curves tend to horizontal.

#### Effects of karst width

The effect of karst width could be considered as effects of L and R, as shown in Fig. 15, the x-coordinate of the tunnel center point is 7, and the upper boundary (U = 1) and bottom boundary (B = 3) are fixed for karst with usual size, the impacts of the left and right boundaries on the central settlement of tunnel floor were studied. From Fig. 15, the shape of the curve is similar to the word ‘Z’. When R < 7, karst has little impact on tunnel floor settlement. With the increasing of R, the settlement gradually increases and finally tends to be stable.

When L ≥ 7 or L < R < 7, these curves are essentially coincident and the values of settlement are all smaller than 1 mm so that the influence of karst on settlement cannot be considered. On the contrary, if L < 7 and R ≥ 7, the impact of karst on settlement is obvious. With the decrease of L, the maximum settlement growing and with the increase of R, settlement tends to stable after increase to the maximum. Finally, in the process of L and R change, when the tunnel floor center is located on the middle of L and R, the settlement at this position is the largest.

#### Effects of rock mass quality

**Figure 16:**
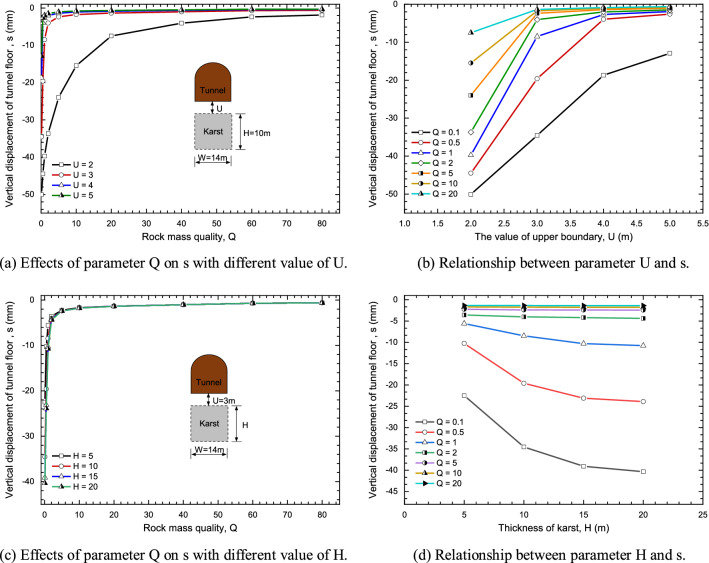
The Effects of rock mass quality Q on the maximum settlement of tunnel floor.

Figure 16a,b shows the variation curve of the rock mass quality with the maximum settlement of tunnel floor when U is 2, 3, 4 and 5, respectively. Also, there is the relationship between the rock mass quality Q and the maximum settlement of tunnel floor when the value of karst thickness is 5 m, 10 m, 15 m and 20 m respectively as presented in Fig. 16c,d.

**Figure 17:**
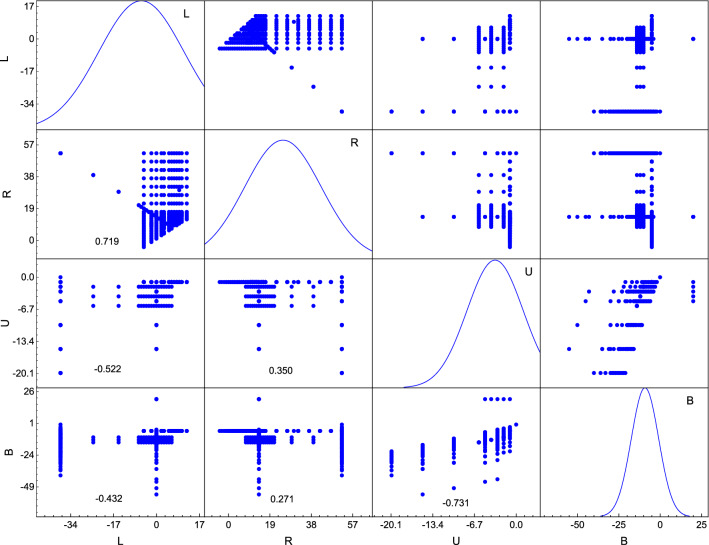
Distributions and correlation coefficients of model input variables in the data set.

According to Fig. 16a, the maximum settlement s of tunnel floor quickly decreases with the increase of rock mass quality Q. Also, with the increase of U, the effects of the rock mass quality Q on the s gradually decreases, as described in Fig. 17b. In addition, it can be found the maximum settlement s of tunnel floor decreases rapidly with the increase of the rock mass quality Q. This shows that under the same conditions of karst boundary, the maximum settlement s of tunnel floor has little affected by the rock mass quality Q in the tunnel site when Q ≥ 5.

Figure 16c,d shows the relationship between the maximum settlement s of tunnel floor and the rock mass quality Q under different H values. It is observed that the maximum settlement s of tunnel floor rapidly decreases with the increase of rock mass quality Q when 0 < Q < 5. On the contrary, when the rock mass quality Q ≥ 5 in the tunnel site, the maximum settlement s of tunnel floor is basically unchanged. In addition, there is little effect on the maximum settlement s of tunnel floor when the thickness of karst H is above 15 m, which is correspond to the results of Fig. 14.

## Predicting results

### Original input variables analysis and prediction models’ parameters

According to the results of influences of Q on s, in the region with Q ≥ 5, the maximum settlement of tunnel floor has been little affect by Q, and the mainly parameters are the karst boundaries including L, R, U and B.

The distributions of four boundaries of karst that were used in the prediction of tunnel floor maximum settlement are illustrated in the diagonal line of Fig. 17. The vertical axis of the diagonal histogram represents the frequency. The upper triangle reveals the pairwise correlation of the model input variables. The correlation coefficients are reported in the lower triangle. Most of the parameters are distributed in a concentrated manner. According to Koo and Li^86^, the r values less than 0.5, between 0.5 and 0.75, between 0.75 and 0.9, and greater than 0.90 are indicative of poor, moderate, good, and excellent reliability, respectively. There was moderate correlation between L and R, U and B, which was due to R is greater than L, and similarly, U is greater than B. There were relatively poor correlations between most input parameters (r < 0.5).

### Optimization of intelligent prediction models

In prediction model, input parameters are the karst boundary of L, R, U and B and output value is the corresponding settlement of tunnel floor from FLAC3D. Of the 456 data sets, 340 patterns were randomly selected as the training data and the remaining 116 data were used for testing. According to the Eqs. (3) to (6), the BPNN model with Bayesian Regularization could be established, and Table 9 presents the connection weights and bias of the optimal network. As for RF model, the hyperparameters and its default value are listed in Table 10.

**Table 9:** Connection weights and bias.

k_i_	w_hki_				w_ok_	b_hk_	b_0_
	1	2	3	4			
1	4.2605	3.4554	3.7972	− 8.1639	2.7255	1.7510	1.9017
2	20.9391	− 0.2385	1.7936	− 6.0430	0.5745	14.2413	
3	3.5439	− 8.9283	− 6.6688	− 0.1692	67.0555	3.5777	
4	3.3531	− 8.8539	− 6.6780	− 0.1178	67.1183	3.4105	
5	0.2498	20.6089	4.6577	17.0069	0.8506	10.7792	
6	6.7630	24.7395	13.5221	1.1696	0.3237	4.5857	
7	7.1056	23.4865	25.4642	4.9186	0.3883	32.4610	
8	9.9134	9.5168	14.3857	2.8671	4.9222	0.2841	
9	11.4950	11.6263	33.6791	11.1526	0.3830	31.0921	
10	4.3978	3.8841	2.0611	5.1418	6.5138	7.5623

**Table 10:** Hyperparameter optimization.

Condition	n_estimators	min_samples_split	max_features
Default	100	2	Auto
Optimal	70	13.0865	2

### Predicting results

**Figure 18:**
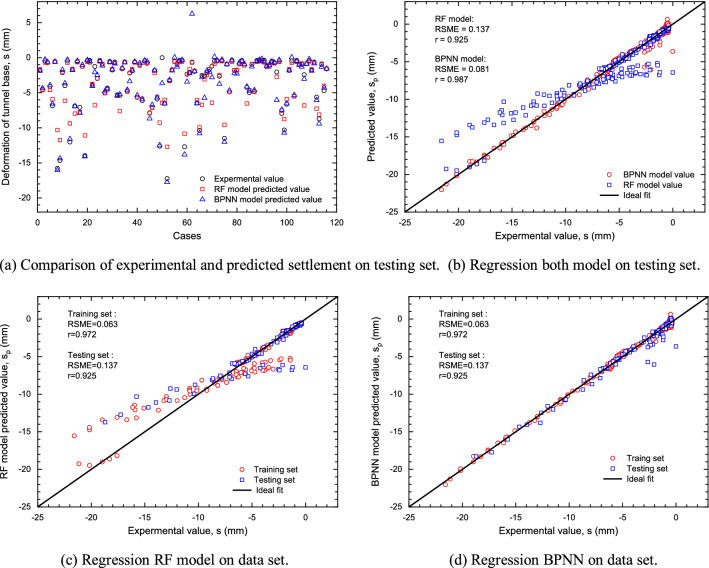
Experimental versus predicted tunnel floor settlement for the BPNN and RF model on data set.

RMSE and r were selected as evaluation indexes. The evaluation of the BPNN and RF models were performed on both training set and testing set as shown in Table 11. The general predictive performance of the BPNN and RF models with optimum hyperparameters on training set was quite acceptable (Fig. 18). Figure 18a provides a visual comparison of predicted and experimental settlement values in testing set, indicating that predicted values are both relatively consistent with experimental values in BPNN and RF. The results of regression analysis of predicted and experimental settlement values in testing set are shown in Fig. 18b. Most points fell around the ideal fitting line, and values of r between the experimental and predicted settlement values of BPNN and RF model were 0.987 and 0.925, respectively. As suggested in previous studies, a model with r values larger than 0.8 can be regarded as acceptable^87,88^. In testing set, although r value of BPNN is 0.987, which is smaller than that of RF model and has great potential for making more reliable predictions. The RMSE of the BPNN and RF model with optimal hyperparameters was 0.081 and 0.137, also indicating that relatively good models had been achieved on the testing set.

**Table 11:** Error comparison between BPNN and RF model.

Predictive model		Correlation coefficient ‘r’	RMSE
BPNN	Training set	0.972	0.016
	Testing set	0.925	0.081
RF	Training set	1.000	0.063
	Testing set	0.987	0.137

The results of regression analysis of predicted and experimental settlement values in both training and testing set are shown in Fig. 18c,d. Most points fall around the ideal fit line, and r values of BPNN and RF model are both larger than 0.9 in training set, which are 0.972 and 1.0 respectively. The RMSE of the BPNN and RF model in training set were 0.016 and 0.063.

### Relative importance of karst boundaries

The tunnel floor settlement under load with different karst spatial distribution was studied by established BPNN and RF models to predict the settlement. The variation interval of each parameter is set to fluctuate up and down within the size range of the numerical model, and the probability distribution of each parameter is assumed to be uniform distribution. The optimal Latin hypercube design is used to sample each parameter, and the sampling results are applied to the prediction model. According to the sensitivity index calculation method based on Sobol method provided by Saltelli^89^, the first-order sensitivity index and total effect index of each parameter of the three prediction models are calculated respectively. The calculation results are shown in Table 12.

**Table 12:** Results of relative importance analysis.

Method	Parameter	First-order sensitivity *S*	Total effect index *S*_*T*_
BPNN	L	0.177	0.756
	R	0.073	0.649
	U	0.039	0.287
	B	0.048	0.319
RF model	L	0.833	0.944
	R	0.017	0.030
	U	0.016	0.057
	B	0.020	0.100

The ability to investigate the relative importance of the influencing variables is another crucial reason why the model is widely used in prediction problems. It can be used to rank the influencing variables according to their contribution to the performance of the prediction model. To reveal the effect of influencing variables on the predicted settlement values of tunnel floor, a sensitivity analysis was conducted for these influencing variables.

Comparing the first-order sensitivity index and total effect index of each parameter in two models as shown Table 12, the first-order sensitivity index and total effect index of L is the largest in the corresponding sensitivity index. In the RF model, the first-order sensitivity index and total effect index of L are significantly greater than the corresponding sensitivity indexes of the other three parameters that means both the influence of a single parameter on the output variance of the model or the total influence of a parameter on the output variance, the change of L has the greatest influence on the settlement of the tunnel floor. Therefore, the small variation range of L is the reason why the prediction result of RF model is not as good as that of BPNN model. In order to improve the prediction effect of RF model on tunnel foundation settlement, the variation range of L should be increased in the model compared with other parameters. In the BPNN model, the first-order sensitivity and total effect index of L are greater than others which is relatively close to the value of R, indicating that L and R has a greater impact on the prediction results of the model.

**Figure 19:**
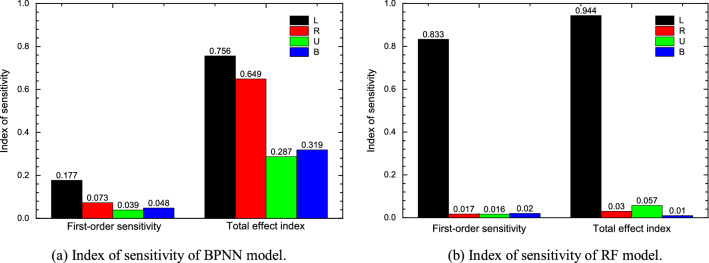
Comparison of sensitivity index between BPNN model and RF model.

As shown in Fig. 19, which can reflect the influence of various parameter changes on the settlement of tunnel floor visually, whether considering the influence of a single input parameter on the model output variance or the total influence of an input parameter on the model variance, L has the greatest influence on the model variance in RF model and BPNN model. The first-order sensitivity and total effect index of each parameter in RF model are relatively close, and the first-order sensitivity and total effect index of L are much greater than the other three parameters. The first-order sensitivity of BPNN model is far less than the total effect index, indicating that the influence of a single parameter on the model variance is far less than that of a parameter considering the interaction of various parameters.

## Conclusion

In this paper, an intelligent displacement prediction model for karst tunnels subjected to railway loads, was developed following the theory of ML and finite difference element modelling. The newly proposed prediction model was then applied to evaluate the settlement of Changqingpo tunnel floor, located in the southeast of Yunnan Guizhou Plateau, China, as a case study. The numerical analysis of the Changqingpo tunnel under railway loads, was performed in the platform of FLAC 3D, in terms of various karst positions and sizes with a total of 456 cases. BPNN and RF models were then used to study the numerical results and then predict the tunnel floor settlement. Conclusions were drawn as follows:

1. Numerical simulations have been validated by the filed data from Yujinshan tunnel, which was belong to the same line of Changqingpo tunnel. Numerical results showed that the karst located at the bottom of tunnel floor had large effects on the settlement of tunnel floor and the settlement increased as monitoring site was near the center of tunnel floor. The maximum settlement happened on the center point of tunnel floor. The karst thickness and the influence of U could be ignored when the value of U is 15 or more, whilst the settlement growing with the increase of karst width and the increase of settlement becomes weak if the width is greater than 30 m.
2. The prediction results by using the BPNN and RF prediction model indicated that relatively good models had been achieved on data set. BPNN held great potential for making more reliable predictions due to r values of BPNN and RF model are 0.987 and 0.925 in testing set.
3. According to first-order sensitivity index and total effect index of two predicting models, whether considering the influence of a single input parameter on the model output variance or the total influence of an input parameter on the model variance, L has the greatest influence on the model variance in RF model and BPNN model. And improving the precision of left boundary could make prediction model more accurate.
4. The dataset in this manuscription is from the results of numerical simulations with the validations of the numerical simulations have been validated due to the limitation of test conditions or field measurement. But the best way is to use field measurement if the construction site is allowed, which can increase the accuracy of predictions and significantly reduce the risk of pre-construction decisions. Meanwhile, for expanding the scope of the prediction model, the factors that influence the tunnel base settlement also should be considered including the water in karst, the filled soil behaviors et al.
